# Bringing synapses into focus: Recent advances in synaptic imaging and mass-spectrometry for studying synaptopathy

**DOI:** 10.3389/fnsyn.2023.1130198

**Published:** 2023-03-15

**Authors:** Nicole Hindley, Anna Sanchez Avila, Christopher Henstridge

**Affiliations:** ^1^Division of Cellular and Systems Medicine, University of Dundee, Dundee, United Kingdom; ^2^Euan Macdonald Centre for Motor Neuron Disease, University of Edinburgh, Edinburgh, United Kingdom

**Keywords:** synapses, imaging, synaptosomes, proteomics, synaptoneurosomes, neurodegeneration, mass spectrometry

## Abstract

Synapses are integral for healthy brain function and are becoming increasingly recognized as key structures in the early stages of brain disease. Understanding the pathological processes driving synaptic dysfunction will unlock new therapeutic opportunities for some of the most devastating diseases of our time. To achieve this we need a solid repertoire of imaging and molecular tools to interrogate synaptic biology at greater resolution. Synapses have historically been examined in small numbers, using highly technical imaging modalities, or in bulk, using crude molecular approaches. However, recent advances in imaging techniques are allowing us to analyze large numbers of synapses, at single-synapse resolution. Furthermore, multiplexing is now achievable with some of these approaches, meaning we can examine multiple proteins at individual synapses in intact tissue. New molecular techniques now allow accurate quantification of proteins from isolated synapses. The development of increasingly sensitive mass-spectrometry equipment means we can now scan the synaptic molecular landscape almost in totality and see how this changes in disease. As we embrace these new technical developments, synapses will be viewed with clearer focus, and the field of synaptopathy will become richer with insightful and high-quality data. Here, we will discuss some of the ways in which synaptic interrogation is being facilitated by methodological advances, focusing on imaging, and mass spectrometry.

## Introduction

There are approximately 86 billion neurons in the human brain and each one estimated to have 1,000–15,000 synapses, meaning the average human brain contains over a trillion synaptic connections (Azevedo et al., 2009; Obi-Nagata et al., 2019). Synapses allow quick responses to motor, behavioral or emotional stimuli and, synaptic plasticity, an activity-dependent process that changes the strength of neuronal networks, facilitates the strengthening and storage of memories. Therefore, when synaptic dysfunction occurs it can induce catastrophic consequences for essential neurological processes. Synaptopathies, characterized by pathological synaptic dysfunction and loss, are now recognized as a common component of many neurodegenerative disorders such as Alzheimer’s disease (AD), Amyotrophic Lateral Sclerosis (ALS), Parkinson’s disease (PD), and Huntington’s disease (HD) (Kerrigan and Randall, 2013; Nishimura and Arias, 2021; Bellucci et al., 2022). Synaptopathy may arise in various ways: for example, mislocalization and aggregation of disease-associated proteins can affect synaptic function leading to breakdown (Lim and Yue, 2015; Wang et al., 2017; Taoufik et al., 2018). Alternatively, alterations in integral synaptic molecular composition may result in protein networks becoming dysregulated, driving synaptic dysfunction, and loss (Ardiles et al., 2017; Torres et al., 2017). Further, aberrant glial-mediated synaptic pruning is increasingly recognized as a driving mechanism of neurodegenerative disease, revealing non-autonomous influencers on synapse health (Cho, 2019). It has been shown that multiple proteins from the complement system, an innate immune response pathway, are elevated in the brains of patients with various neurodegenerative diseases and this pathway may be a major player in glial phagocytosis of synapses (Hong et al., 2016; Gomez-Arboledas et al., 2021; Fatoba et al., 2022).

Growing evidence suggests that structural and functional dysregulation of synapses is one of the earliest pathological events in neurodegeneration (Henstridge et al., 2019). Stopping or reversing synaptic dysfunction could help slow down or halt disease progression, making synapses an attractive target for further study, and therapeutic interventions. For example, recent work studying synthetic organizer proteins has shown they can potentially restore synaptic function in neurodegenerative diseases through interaction with presynaptic cell adhesion proteins and postsynaptic α-amino-3-hydroxy-5-methyl-4-isoxazolepropionic acid (AMPA) receptors. This encouraged excitatory synapse formation both *in vitro* and *in vivo* and resulted in improved motor coordination in models of spinal cord injury and improved memory in mouse models of AD (Suzuki et al., 2020). Having the capacity to intervene and preserve or potentiate synaptic function could result in a new wave of treatments for diseases in which synaptopathy is a central feature.

However, synapses are not easy structures to study. Their vast numbers, small size, and structural delicacy means that developing and utilizing techniques to investigate synapse structure and molecular composition can be difficult. In this review, we will discuss various methods used to study synapses, both at the structural and molecular level, outlining their advantages and limitations to help paint a picture of how synapses can be studied, and how these methods can be implemented in individual studies. We will also discuss advancements made using these techniques in the field of neurodegeneration to understand more about synaptopathies and their role in neurodegenerative diseases. Using robust techniques to study synapses is pivotal in the aim of developing potential future therapeutics for people living with neurodegenerative disease.

## Imaging synapses in intact tissue

Despite the challenges that imaging synapses poses, modern technologies now allow us to study various aspects of synaptic structure and composition from a whole brain level, down to subsynaptic resolution, using different imaging approaches. Here, we will start by discussing some of the techniques developed for human synaptic analysis, from *in vivo* whole-brain imaging to higher resolution imaging of post-mortem tissue. We will then explore other new imaging approaches that have been only used in model systems to date but show great potential for understanding human disease.

### *In vivo* study of human synapses

A rapidly developing area is the *in vivo* study of synapses in the human brain. This is a reasonably non-invasive technique that can provide insight into disease progression and how synapses correlate with phenotypic change. This could highlight synaptic targets at early disease stages, as well as serve as a useful monitoring tool of disease progression in clinical trials.

*In vivo* synaptic density can be imaged by positron-emission tomography (PET) scanning of the brain after exposure to a radioligand coupled to a synaptic marker–for instance, synaptic vesicle glycoprotein 2 (SV2) (Finnema et al., 2016; Cai et al., 2019). This technique is proving insightful and could shine a light on dynamic changes in synaptic density at different stages of disease. However, there are still some challenges with this technique, such as generating robust, reliable, and specific markers that can cross the blood-brain barrier and achieving accurate segmentation of whole brain scans into anatomical areas of interest. Still, there are now several publications using this technique in the context of neurodegenerative diseases. For instance, SV2 PET scanning has been used to study synaptic density loss in the hippocampus (Chen et al., 2018), and whole brain (Mecca et al., 2020) of AD patients as well as the association between amyloid plaques and synaptic density (O’Dell et al., 2021). Similarly, studies in Parkinson’s, have used this technique to confirm synaptic loss in the substantia nigra, red nucleus and locus coeruleus, the main affected areas in PD (Matuskey et al., 2020) as well as to study overall cerebral synaptic density in primary tauopathies (Holland et al., 2022). In ALS, ^18^F-SynVesT-1 PET (another SV2 radiomarker) was used to assess synaptic density changes in different areas of the brain (Tang et al., 2022) and importantly, the results closely aligned with previous post-mortem data (Henstridge et al., 2018). Moreover, SV2A PET has also been used in frontotemporal dementia (FTD), which is thought to exist on a disease spectrum with ALS, to study synaptic density changes in patients with behavioral variant FTD (Malpetti et al., 2023), and in carriers of a repeat expansion in the chromosome 9 open reading frame 72 (*C9ORF72*) gene, which has been linked to both ALS and FTD (Malpetti et al., 2021). This exciting work revealed region-specific synaptic change that associates with cognitive change and genotype. As the utility of this approach widens, there is interesting recent evidence that synaptic PET imaging in the spinal cord is possible (Rossano et al., 2022), which will be important for diseases like ALS as well as understanding spinal cord injury and monitoring regeneration.

It is clear that the real-time imaging of synapse density in the living human brain will have a huge impact on our understanding of synapse changes in disease. However, issues surrounding tracer specificity, sensitivity, and whole brain image analysis at the millimeter scale need to be refined to improve the accuracy of this approach.

### Post-mortem study of human synapses

**FIGURE 1:**
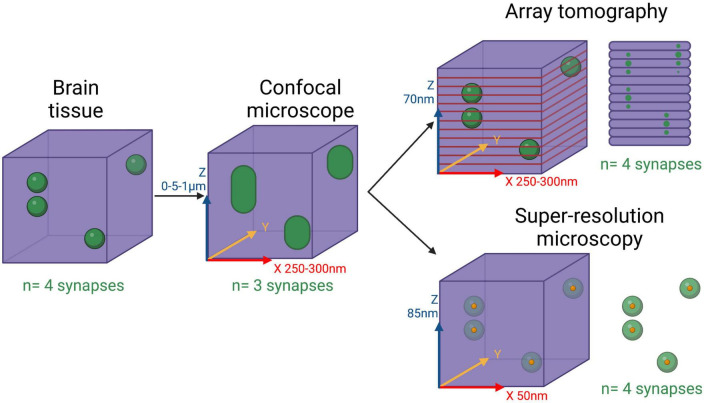
Limitations of confocal microscopy for 3D synaptic imaging. Diagram showing 4 synapses in a 3-dimensional piece of brain tissue and how different imaging approaches would resolve them. Confocal microscopes have a diffraction-limited resolution in all axis, meaning two synapses in close proximity will be observed as just one large entity. To solve this problem, array tomography resolves all the synapses by cutting the tissue very thin (70 nm) thus setting the axial resolution by the thickness of the sections. 3D super-resolution single molecule light microscopy is able to pinpoint all synapses by carefully identifying the central point of each synapse and highlighting them separately. Resolutions achieved by the different techniques are shown in the axis. Created with BioRender.com.

For more detailed synaptic imaging at a greater resolution than the *in vivo* techniques described above, we need to use post-mortem tissue. This approach allows the use of advanced imaging techniques to gain nanometer resolution, but generally limits the study to end-stage human disease. Another challenge is that synapses are smaller than the axial (z-plane) resolution limit of normal light microscopy. This means that if synapses are very close to each other – and given their density in the brain it is likely they would be – light microscopes would often see them as one large object rather than two or more separate entities (Figure 1). Therefore, researchers have invested a lot of effort over several decades to develop ways of breaking the light diffraction limit and achieve super-resolution detail.

### Electron microscopy

Historically, the most common approach to study synaptic structure has been electron microscopy (EM). Electron microscopes break the light diffraction limit in the 2-dimensional planes by using a controlled beam of electrons instead of photons of light, achieving resolutions of less than a nanometer. There are two main modalities of EM, transmission electron microscopy (TEM), in which the beam of electrons travels through ultrathin tissue sections providing a 2D monochrome image, and scanning electron microscopy (SEM), where the electrons are detected after bouncing off the sample, resulting in a 3D image of the surface of the sample (Titze and Genoud, 2016). Heavy metals such as lead are often used in EM to increase the contrast in the sample and allow for accurate structure observation.

Electron microscopy is still considered the gold standard imaging technique for studying synaptic structure and has been used to study synapse density in Alzheimer’s disease since the seminal work by DeKosky and Scheff (1990) and Scheff et al. (1993). More recently, it has been used to assess the hallmark neurofibrillary tangles and amyloid deposits and their impact on synapses (Katsuse et al., 2006). Exploiting the exquisite detail obtained by EM, recent work has highlighted brain region specific changes in the synaptic accumulation of mitochondria in human AD brains compared to controls (Pickett et al., 2018). EM has also proved useful in revealing early evidence of synapse loss in human ALS and highlighting altered synaptic ultrastructure and immaturity (Sasaki and Maruyama, 1994a,b; Sasaki and Iwata, 1996a,b; Qiu et al., 2014; Henstridge et al., 2018; Deshpande et al., 2019).

Despite the unquestionable power of EM when it comes to understanding synapse structure and density, it is a difficult technique to master and requires highly specialized microscopes. One issue that can have a dramatic impact on image quality is difficulty retaining tissue structural integrity during sample processing. One approach to retain integrity is to use cryo-electron tomography (cryo-EM). Flash-freezing samples at very low temperatures can improve structure preservation and by utilizing this tissue processing approach, cryo-EM can achieve incredible resolution of protein structure, down to 3.5 Å. This technique can easily achieve the resolution required to study synaptic structure, however, given its highly technical nature, only a few research teams have mastered it (Gopalakrishnan et al., 2011; Liu et al., 2020). Still, cryo-EM has been used to study the structure of aggregation-prone proteins commonly found in diseased brain. For example, it has been used to study the ultrastructure of tau filaments from the brain of an AD patient, revealing how different isoforms of tau aggregate (Fitzpatrick et al., 2017). Cryo-EM has also been used in ALS to study the ultrastructural composition of reversible and irreversible TAR DNA binding protein 43 (TDP-43) aggregates, a hallmark of the disease (Cao et al., 2019). It has also been used to describe the structure and composition of dipeptide repeat aggregates, characteristic of *C9ORF72*-ALS – the most common genetic form of ALS–and the authors showed aggregates can recruit proteasomal subunits, pointing to a potential pathological mechanism (Guo et al., 2018). Cryo-EM has also been crucial in PD, revealing the ultrastructure of aggregation prone α-synuclein inclusions (Guerrero-Ferreira et al., 2019; Boyer et al., 2020), which could shine a light into potential therapeutic approaches to prevent aggregation.

A variation of electron microscopy, immuno-EM, adds detail to EM images by using antibodies with electron-dense gold particles attached. This allows for specific labeling of proteins of interest, which is useful for providing detail on protein localization at the nanometer scale. This approach has been widely used to accurately assess the synaptic and subsynaptic localization of key synaptic proteins, and in skilled hands, it is possible to use different sizes of gold particles to label different proteins in the same sample (Nusser et al., 1994; Matsubara et al., 1996; Petralia et al., 1998, 2001; Xiao et al., 1998; Valtschanoff and Weinberg, 2001; Paik et al., 2021; Petralia and Wang, 2021). In the context of disease, immuno-EM has been used to characterize α-synuclein as the main component of Lewy Bodies (Spillantini et al., 1997), characteristic of PD and dementia with Lewy bodies, and that these are present in the substantia nigra of PD patients (Crowther et al., 2000). It was also used to reveal that perisynaptic protein complexes are discretely disrupted in Fragile X-syndrome, which is impactful enough to pathologically impair synaptic function (Jung et al., 2012). However, a drawback of this approach, discussed in Kay et al. (2013), is the fact that immuno-EM techniques can be thwarted by low antibody penetration or epitope access, which makes it challenging to use EM for studies of synaptic protein composition. However, several comprehensive reviews describe optimization approaches one can take to increase antibody penetrance and specificity to help circumvent these issues (Phend et al., 1992; Petralia and Wang, 2021; Tao-Cheng et al., 2021).

Clearly, EM–with all its modifications discussed above–is a powerful technique that has provided significant insight on synapse structure and density changes in health and disease. However, electron microscopy is a time-consuming imaging approach that generally allows the study of a small number of synapses per sample due to the extremely small images captured, therefore other approaches that can provide a similar level of detail but in a more high-throughput manner would be preferable.

### Array tomography

Array tomography (AT) is one imaging approach that can overcome some of the difficulties faced with EM (Sanchez Avila and Henstridge, 2022). Developed in 2007, (Micheva and Smith, 2007) and later adapted for human post-mortem tissue (Kay et al., 2013), this high-resolution imaging technique allows a detailed study of the synapse at both a density and protein composition level. Array tomography overcomes the axial resolution limit of light microscopy by physically cutting ultrathin (70 nm) serial sections of resin embedded tissue (Figure 1). Sections are immuno-stained and imaged using widefield fluorescence microscopes. The images are then stacked and rendered into a 3D model. This approach is advantageous over EM as the microscopes are more easily accessible and the technique and analysis is generally less time consuming. Another advantage is the opportunity for multiplexing, as ribbons can be stripped and re-stained. This allows the analysis of multiple proteins within individual synapses and has been validated for up to six stripping rounds, allowing for the identification of 18 different synaptic markers (Kleinfeld et al., 2011). Array tomography has now been widely used as a high-resolution imaging technique to study synapses in several contexts such as Alzheimer’s disease (Koffie et al., 2012, 2009; Tai et al., 2012; Spires-Jones and Hyman, 2014; Hesse et al., 2019; Pickett et al., 2019, 2016; Querol-Vilaseca et al., 2019; Rupawala et al., 2022), dementia with Lewy bodies (Colom-Cadena et al., 2017, 2013; Rupawala et al., 2022) and ALS (Henstridge et al., 2018). Collectively, these studies (and others) have highlighted changes in synaptic density, molecular composition, and the aggregation of disease-associated proteins in the synapse. Thus, AT is an established technique that allows for a high throughput study of synapses and the multiplexing of several proteins of interest.

### Super-resolution microscopy

While AT uses physical dissection to improve image resolution, another approach is to exploit super resolution microscopy techniques (Eisenstein, 2015; Lambert and Waters, 2017; Raduloviæ et al., 2021; Fuhrmann et al., 2022) such as stochastic optical reconstruction microscopy (STORM, Rust et al., 2006), stimulated emission depletion (STED, Hell and Wichmann, 1994), photoactivated localization microscopy [PALM, (Betzig et al., 2006)], and structured illumination microscopy (SIM, Gustafsson, 2000). Briefly, these super-resolution imaging techniques achieve low nanometer resolutions by utilizing different ways to localize fluorophores more accurately in a sample, for instance by switching the fluorescent state of single molecules one at a time and determining their precise localization or by using two different lasers in a donut formation to selectively switch some fluorophores on while depleting others around them. These techniques have been used on their own (Masch et al., 2018; Böger et al., 2019), or in combination (Broadhead et al., 2016; Crosby et al., 2019) to study synapses (Jones et al., 2017) as well as disease relevant structures, such as amyloid plaques (Querol-Vilaseca et al., 2019) in human post-mortem tissue. These approaches have been available for many years now but are still relatively limited in use due to expensive custom setups required and extensive user training. A more detailed explanation of these approaches as well as their potential application in the field of neuroscience, can be found in other excellently written reviews (Sigrist and Sabatini, 2012; Tønnesen and Nägerl, 2013; Padmanabhan et al., 2021).

## Imaging synapses in disease models

While this review largely focuses on the study of human synapses, we will briefly explore recent imaging techniques that have only been used to date in models of neurodegeneration but show tremendous potential if applied to human studies.

### In vivo

As previously discussed, there is very valuable information to be gained from *in vivo* study of human synapses. However, there are imaging approaches that for various ethical and practical reasons cannot be utilized in humans and so we must rely on the use of model systems.

One simple yet elegant approach for *in vivo* synaptic imaging in mouse models is the use of cranial windows to gain imaging access to superficial layers of the cortex. Researchers can fill neurons with fluorescent molecules such as Green fluorescent protein (GFP) *via* intracortical injection or genetic modification and longitudinally image individual spines using multiphoton imaging. This technique also allows for the study of dendritic spine density and dynamics and has been used in murine models of AD to study the toxic effects of amyloid-β on spines near amyloid plaques (Spires et al., 2005; Wu et al., 2010).

Super resolution imaging has also been adapted for *in vivo* analysis. STED has been performed in live mice and has provided exquisite synaptic detail from both the cortex and hippocampus (reviewed by Fuhrmann et al., 2022). Moreover, techniques such as PALM can be taken one step further to perform what is known as sptPALM, which allows single-particle tracking in *in vivo* models; this is still a relatively new approach and is limited by the need for models to be transparent enough to image *in vivo*, but it has been used to track syntaxin -1A at the motor nerve terminal of a *Drosophila* larvae (Bademosi et al., 2018).

### In vitro

Super resolution imaging can also be performed on cultured neurons. For instance STORM has been used to study the synaptic localization of FUS, a protein of interest in ALS, in cultured rat hippocampal neurons and human iPSC-derived motor neurons (Deshpande et al., 2019). SIM was used to study the synaptic localization of Bin1, an Alzheimer’s-associated protein and both STORM and STED were used to study the synaptic localization of γ-secretase, the protease that cleaves the AD-associated β-amyloid (Schedin-Weiss et al., 2016).

### Post-mortem

Array tomography and EM can also be combined in what is called conjugate AT. This approach has only been used for studying rodent brain to date, but shows great potential for advancing our understanding of human disease.

In conjugate AT, serial sections are taken of the tissue embedded in either resin or plastic. Then, the sections are immuno-stained and imaged as per conventional AT protocols. Afterward, the ribbons are washed and prepped for EM. This technique combines the strengths of EM and AT, conserving as much of the ultrastructure as possible as well as allowing multiplexing of different proteins (Collman et al., 2015; Bloss et al., 2018). AT has also been performed on physiologically characterized synapses (Valenzuela et al., 2016; Holderith et al., 2020; Micheva et al., 2021). Combining physiological and molecular analyses at a single-synapse level in this way will undoubtedly reveal important insight into physiological and pathological synaptic function.

Super resolution imaging studies in the context of neurodegeneration have also mostly used disease models. STED has revealed, in a *Drosophila* larvae model of *FUS*-ALS, the structural degeneration of neuromuscular junctions (Shahidullah et al., 2013). These techniques can also be combined with AT to provide super-resolution in all three dimensions (Querol-Vilaseca et al., 2019; Kim et al., 2021). However, despite the undeniable power of these super-resolution imaging approaches, they require specialized equipment and highly experienced users, currently restricting their mainstream use.

There are several new imaging and sample processing developments that may facilitate the analysis of synaptic biology. For example, the recently published SEQUIN imaging approach (Sauerbeck et al., 2020). SEQUIN (Synaptic Evaluation and QUantification by Imaging Nanostructure) combines image scanning microscopy and localization-based analysis. The authors used SEQUIN to analyze the synaptic density in three different mouse models of Alzheimer’s disease, and reveal synapse loss near amyloid plaques, replicating previous literature (Koffie et al., 2009). This technique is reasonably accessible, and its high throughput ability would be advantageous over techniques such as EM. Moreover, it has the capacity to be combined with other techniques for the study of physiologically characterized synapses as well as adaptation for *in vivo* use, making it a potentially excellent resource for future synaptic studies.

Lastly, another recent approach one can use is expansion microscopy. This technique involves physically expanding the sample by using a polymer that swells in the presence of water (Chen et al., 2015), meaning the nanometer scale expands to the micrometer scale making to possible to gain super-resolution detail without the need of specialized super-resolution microscopy setups. Expansion microscopy has now been used in a variety of tissues and model systems to reveal nanometer-scale information (Gallagher and Zhao, 2021). Some recent work has developed this approach to study synaptic structure and amyloid fibrils, and the authors terming the optimized technique “expansion revealing” (Sarkar et al., 2022). This sample processing technique may help make the analysis of synaptic biology easier and more accessible, by simply making them bigger.

## Imaging summary

There are multiple imaging methods to consider when studying synaptic structure, and the appropriateness of each technique will vary depending on the aims of the study and the problem being addressed. However, imaging approaches have the obvious advantage of providing visual data on the structure and protein composition of the synapse and, as techniques improve and become more accessible, we will surely see increased uptake in neurodegenerative research.

The powerful imaging techniques discussed allow us to investigate synaptic structure, composition, and loss of synaptic density at the whole brain level and fixed brain tissue sections, providing insights into structural synaptic changes in neurodegeneration. However, the full pathological underpinnings of synaptopathies in neurodegeneration are yet to be fully understood and we cannot delve further into the mechanisms behind synaptopathies through imaging techniques alone. To do this, we need to reduce synapses down to their molecular composition and identify changes in synaptic protein expression, from which bioinformatic analyses can reveal dysregulated pathways that may be contributing to synaptic dysfunction.

## Studying synapses at the molecular level

### Generating synaptic samples

There are several protocols that allow us to go from whole brain tissue down to a synaptically enriched fraction, and through this process that we can start to access the molecular composition of synapses. First, fresh frozen brain tissue is homogenized in isotonic solution to lyse the tissue, and then the homogenate undergoes either a series of ultracentrifugation or filtration steps to isolate the synapses. Through ultracentrifugation, synaptosomes are generated, described in 1964 as “thin-walled bags containing cytoplasm packed with synaptic granules or vesicles, frequently one or more mitochondria are also present” (Whittaker et al., 1964). Synaptosome preparation protocols vary slightly but generally all follow homogenization and ultracentrifugation steps (Gulyássy et al., 2020). Through this process, synaptic terminals are detached from their axons and their membrane reseals, creating a membrane-bound presynaptic terminal sac containing synaptic vesicles and mitochondria. The post synaptic component of a synaptosome consists of a segment of the postsynaptic membrane and spine with the post synaptic density (PSD) still intact and connected to its outer surface (Weiler, 2009). Synaptosomes are relatively homogenous and maintain many synaptic metabolic and enzymatic activities (Whittaker et al., 1964; Ahmad and Liu, 2020). Some synaptosome protocols have been expanded to incorporate synaptosome sorting *via* fluorescence, Fluorescence Activated Synaptosome Sorting (FASS). FASS is a sorting method that results in ultrapure synaptosomes, almost free of other non-synaptic components, by utilizing mouse lines genetically modified to express fluorescent synaptic proteins. This protocol has been used recently to define commonalities and differences of the synaptic proteome across a range of brain regions and cell types (Biesemann et al., 2014; van Oostrum et al., 2023). Synaptosomes have been widely used as an *ex vivo* model across to address many diverse topics of synaptic biology (Velásquez et al., 2017; Mallozzi et al., 2018; Plum et al., 2020; Sapp et al., 2020; Kumar et al., 2022). Recently, protocols have been developed that enable the isolation of synaptosomes from human iPSC-derived neurons, incorporating steps to also generate PSD-enriched fractions (Rajkumar et al., 2023). This approach combined with the power of patient-derived cells will undoubtedly increase our knowledge of synaptic change in human disease.

Despite synaptosomes being widely used as an *ex vivo* model, there is increasing evidence to support the idea that synapses consist of more than just pre- and postsynaptic terminus (Chung and Barres, 2012; Henstridge et al., 2019; Morizawa et al., 2022). The tripartite synapse acknowledges the important role that glial cells play in synaptic function: astrocytes, microglia, and oligodendrocytes support synapses within the central nervous system (CNS), and Schwann cells support the neuromuscular junction (NMJ) in the peripheral nervous system (Perea et al., 2009; Petrov et al., 2021). Glia have roles crucial for proper synaptic function, including clearance and reuptake of neurotransmitters, Ca^2+^ homeostasis, receptor distribution, phagocytosing synaptic material, and synaptogenesis (Murphy-Royal et al., 2017; Um, 2017; Kono et al., 2021; Shan et al., 2021; di Benedetto et al., 2022). Neuron-glial interactions have also been shown to participate in synaptic formation, development, and plasticity (Kim et al., 2020). Furthering this, the tripartite synapse has been extended to the tetrapartite synapse described in 2011. The tetrapartite synapse integrates a fourth component to the equation of synaptic function: the extracellular matrix (ECM). Important for synaptic transmission, the ECM can store and consolidate neuronal and glial processes on the molecular level and assists synaptic plasticity through modulation of pre- and postsynaptic receptors and ion channels (Dityatev and Rusakov, 2011).

**FIGURE 2:**
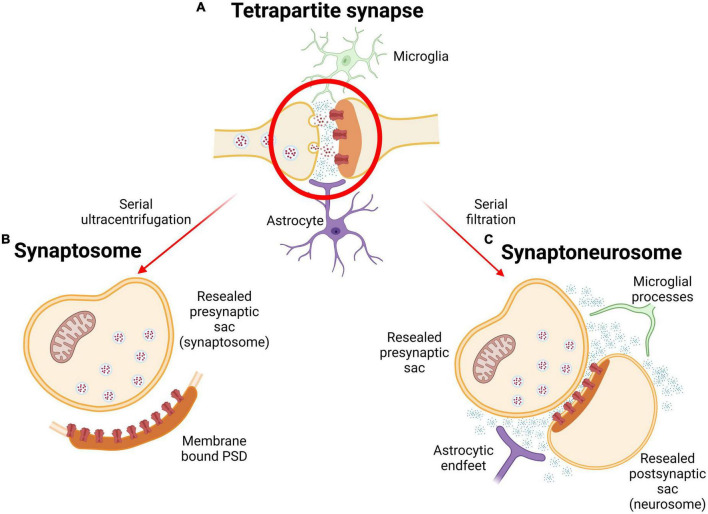
Subcellular isolation of synapses. **(A)** Depiction of the tetrapartite synapse including glial influences and ECM (blue dots) in the extracellular synaptic space. **(B)** Synaptosome produced through gradient ultracentrifugation. **(C)** Synaptoneurosome (SNS) contains microglial processes and astrocytic endfeet as well as EMC molecules, and represents a “crude” synaptic fraction. Created with BioRender.com.

Considering this complex composition, the use of “crude” synaptic fractions, has been argued to be more biologically relevant than synaptosomes. Thus, many studies utilize synaptoneurosomes, or “synaptically enriched” fractions. Produced *via* a series of filtration steps using nylon filters, synaptoneurosomes consist of a resealed presynaptic sac, a resealed postsynaptic spine, intact PSD and glial processes, as well as components of the ECM (Figure 2). Synaptoneurosome studies can therefore reveal changes in glial proteins which, given their significant role in synaptic processes and contribution to neurodegeneration, are important to understand. Not only do synaptoneurosomes present biological advantages, technical advantages such as higher final protein yield with lower amounts of tissue suggest additional time and cost benefits (Hollingsworth et al., 1985; Johnson et al., 1997).

Deciphering the specific location of a protein within the synapse is impossible from a homogenized synaptic sample, but can be achieved by further subcellular fractionation, which separates the pre- and postsynaptic components of the synaptosome or synaptoneurosome sample. Through a series of ultracentrifugation steps on a sucrose density gradient, the pre- and postsynaptic terminals can be separated and analyzed *via* mass spectrometry (MS) or western blot to identify the localization of specific proteins. This is beneficial when the project aims are to identify the subsynaptic location of a specific protein, such as pathological tau in AD (Tai et al., 2012; Bermejo et al., 2014).

Taking this a step further, a modified synaptosome protocol was developed to extract a presynaptic vesicle-enriched fraction. The authors used this approach to reveal that amyloid precursor protein (APP), which is cleaved to produce pathological Amyloid-beta (Aβ) in AD, was present in rat synaptic vesicles, providing extremely detailed information on the subsynaptic localization of this disease-relevant protein (Groemer et al., 2011).

### Proteomics

Once synapses have been isolated from brain tissue in the form of synaptosomes or synaptoneurosomes, methods can be used to determine the synaptic proteome. Proteomics is the study of all the proteins expressed by a biological system, tissue, cell, or structure, such as a synapse. Proteomics is considered a particularly insightful “-omic” approach, as proteins provide a more accurate foundation to interpret biological processes– their presence indicates a functional biological role whether physiological or pathological, as opposed to transcriptomics for example, where some RNAs identified may be degraded before being translated into functional proteins. Nonetheless, transcriptomic studies can reveal important information about synapses and synaptopathies. A recent transcriptomics study investigated synaptic translation behavior using synaptosomes and provided evidence of protein synthesis at excitatory presynaptic boutons, thus exposing the translational ability of presynaptic terminals (Hafner et al., 2019). Despite the interesting insights that transcriptomics can provide into the synaptic translation, proteomics is still arguably considered a more biologically insightful approach when studying synaptopathies and the pathological processes underlying them. Thus, proteomics in neuroscience (“neuroproteomics”) is being increasingly implemented, due to the wide coverage capabilities and the functionally accurate data generated. Further, neuroproteomics can also be used to identify potential biomarkers or potential therapeutic targets within a pathological molecular process (Hasin et al., 2017; Kline et al., 2022).

**FIGURE 3:**
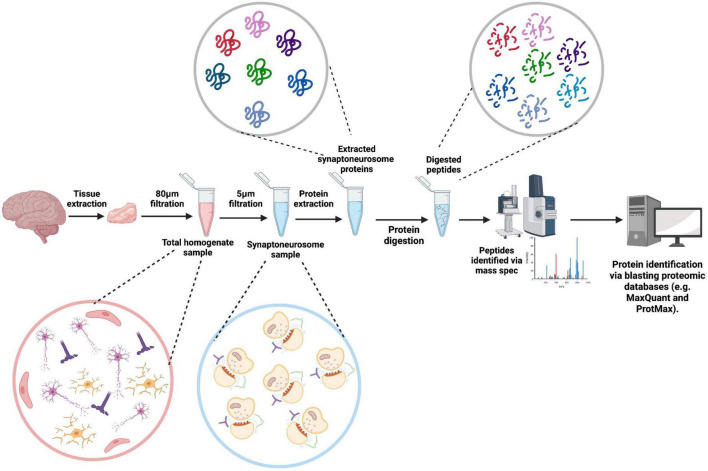
Synaptoneurosome proteomics workflow. A tissue sample is extracted from post-mortem brain tissue, homogenized in homogenization buffer breaking apart the tissue. The sample is then pushed through an 80 μm filter, removing any tissue components, leaving a total homogenate sample containing fragmented cellular components. Further filtration through a 5 μm filter removes all nuclear components of the sample, leaving synaptoneurosomes. Synaptoneurosome proteins are then accessed through protein extraction with a lysis buffer and digested into peptides. The peptides are inputted into the mass spectrometer and identified peptides are blasted against proteomic databases to identify the corresponding proteins. Created with BioRender.com.

Mass Spectrometry (MS) is the most common method used in proteomic studies. Here, molecules within a sample are ionized and the mass-to-charge ratio (m/z) is measured, and the identified peptides used to determine parent proteins by blasting against proteomic databases such as MaxQuant and ProtMAX (Figure 3). Prior to MS, all the proteins in a sample are digested enzymatically [bottom-up MS (BU-MS)] for an unbiased proteomics approach, or specific proteins are selected beforehand for MS analysis (targeted proteomics). Investigations of the proteome generally adhere to BU-MS due to its unbiased nature, ability to detect peptides as small as 0.8–3kDa and initiative to follow the data. All are important considerations when aiming to identify small synaptic proteins and perform a deep characterization of the synaptic proteome (Timp and Timp, 2020).

Traditionally, the most common MS approach has been data-dependent acquisition (DDA) MS, in which peptides with this highest abundance in the sample are identified. This occurs through the selection of the peak spectra in the MS1 phase–only the peptides from the highest peak spectra will be further fragmented and analyzed in the MS2 phase. This approach has been favorable in many proteomic studies of the synapse as it allows the incorporation of tagging, such as isobaric tags used in Tandem Mass Tagged Liquid Chromatography MS (TMT-LC-MS/MS). Tagging enables a multiplex investigation of (currently) up to 16 samples in one MS experiment, which not only reduces technical variance, but also results in a lower demand on equipment and computational resources, reducing the experimental cost. Thus, many researchers utilize the multiplexing capabilities of this MS approach, including recent proteomic studies investigating neuroinflammation at the human and mouse AD synapse using synaptosomes (Wijasa et al., 2020).

Recently, however, there has been a shift to data-independent acquisition (DIA). Unlike, DDA MS, DIA identifies all the peptides present in a sample, by selecting spectra in sequential time windows in the MS1 phase regardless of peak size. This means all peptides present in a sample will be further fragmented in MS2 and taken forward for analysis, not just those with the highest abundance as is the case in DDA. Due to this, DIA allows more proteome coverage with less bias. DIA also requires study-specific spectral libraries for data processing. Studies lacking such libraries such as analysis of post-translational modifications or subcellular compartments will require libraries to be generated prior. This spectra library availability is important to consider prior to DIA MS, as significantly more sample and instrument time is required if there is a need to generate a study-specific library, this increasing costs (Krasny and Huang, 2021). Regardless of the approach taken, synaptoneurosome-based mass-spectrometry analyses can identify around 5,000–6,000 proteins, providing the researcher with an extremely deep coverage of the proteome.

Specific to the synaptic proteomics field, Gajera et al., 2019 developed an adaptation of mass cytometry MS and synaptosome protocols, which they termed “mass synaptometry,” to study the protein content of individual synapses. Mass synaptometry combines synaptosome protocols with cytometry by time-of-flight mass spectrometry (CyTOF), a method that uses antibody probes bound to metal ions to analyze the proteomes of individual cells. The use of metal ions rather than fluorescent tags, as is used in flow cytometry, is beneficial as many antibodies can be combined in a single sample whilst reducing spectral overlap that occurs when using fluorescence. Cell autofluorescence can also be avoided by employing this method (Nowicka et al., 2017). The adaptation of CyTOF to mass synaptometry results in a high-throughput study of individual synaptosomes, which could further reveal how synaptopathies progress in neurodegeneration, such as the prion-like spread seen in tauopathies (Robbins et al., 2021).

Changes in protein expression levels may not necessarily reveal the full picture of molecular change at the synapse. For example, protein-protein interactions are important to identify so we can try to understand how these may change in disease and what the consequences might be on synaptic function (LaCava et al., 2015). Immunoprecipitation MS (IP-MS) can identify interacting proteins by performing a conventional antibody-based immunoprecipitation of a target protein, followed by MS identification of the proteins pulled out in the bound protein complex. A recent IP-LC-MS/MS study of synaptosomes demonstrated that it is possible to identify the co-immunoprecipitated protein partners of SNAP-25, to help focus specifically on SNARE protein changes in AD (Brinkmalm et al., 2014). Despite IP-MS traditionally being the main method to study and identify protein-protein interactions, recent years have seen an increased implementation of TurboID biotin labeling. This method employs biotin ligase, a promiscuous enzyme, to indiscriminately tag neighboring proteins with biotin which can then be extracted and identified by mass-spectrometry (Strack, 2018; Marcassa et al., 2023). This proves much more sensitive than IP-MS and has been used to identify tripartite synaptic connections between astrocytes and neuronal synapses (Takano and Soderling, 2021). Similar methods can be used with ascorbate peroxidase-derived (APEX2) or horseradish peroxidase (HRP), peroxidase enzymes that are more suited to *ex vivo* models and have been used to investigate dopaminergic synaptosomes, and the presynaptic proteome (Hobson et al., 2022).

Following any type of proteomic experiment, data collected must then be analyzed, and interpreted biologically using various bioinformatic analyses (Chen et al., 2020). Enrichment analysis is insightful for identifying enriched GO terms within a data set, this can be done using software such as DAVID (Database for Annotation, Visualization, and Integrated Discovery), g:Profiler and ShinyGo (Raudvere et al., 2019; Ge et al., 2020; Sherman et al., 2022). Pathway analysis enables identification of molecular pathways that involve proteins within the dataset, software such as REACTOME, KEGG, and Ingenuity Pathway Analysis are commonly used for proteomic pathway analysis (Kanehisa and Goto, 2000; Krämer et al., 2014; Gillespie et al., 2022). In terms of synapse specific bioinformatic analysis, SynGO can be used to reveal subsynaptic localization of your identified proteins (Koopmans et al., 2019).

Interestingly, whole tissue homogenates have also been used in neuroproteomic studies and many have cemented the importance of synaptic protein changes in neurodegenerative diseases. For example, a recent LC-MS/MS proteomics study identified synaptic markers of cognitive decline in post-mortem AD homogenates and described a correlation between the loss of certain synaptic proteins and the extent of cognitive impairment (Bereczki et al., 2018). Synaptic proteins were also recently shown to be significantly altered in ALS-FTD whole tissue homogenates from human cortex (Umoh et al., 2018). These studies show that even in a very complex sample, changes in synaptic proteins and processes, even without isolating synapses prior, can be detected reinforcing the notion of synaptopathy playing a significant role in disease.

### Experimental considerations

The use of synaptosomes or more complex synaptoneurosomes is an important choice to consider, as subsequent pathway analyses will be influenced by the biological content of the sample. For example, a recent TMT-LC-MS/MS study of synaptoneurosomes generated from human AD cortex, revealed an enrichment of immune-related pathways (Hesse et al., 2019). Another recent TMT-LC-MS/MS analysis of synaptoneurosomes from ALS cortex, found complement, and coagulation cascades were highly enriched in ALS patients with cognitive decline (Laszlo et al., 2022). These findings may have only been identified due to glial processes being present in synaptoneurosomes. This is important because glia are known to utilize the complement cascade at the synapse to instigate phagocytosis of synapses, which is known to be increased in neurodegeneration (Henstridge et al., 2019; Dalakas et al., 2020; Liu et al., 2023). This highlights the importance of considering the physiological differences between synaptosomes and synaptoneurosomes when investigating dysregulated pathological synaptic pathways.

Proteomic approaches can be extremely useful ways to review the molecular landscape of synaptic pathology. Synaptosomes and synaptoneurosomes are robust and relatively easily produced *ex vivo* models which can be produced from cultured cells, animal tissue, and human post-mortem brain, allowing the researcher to focus specifically on synaptic protein content. However, the data needs to be validated, preferably in intact tissue. This shows an important interplay is required between molecular and imaging approaches to truly uncover the important aspects of synaptic change in disease.

## Conclusion

Overall, with the increasing awareness that synaptopathy is one of the first pathological events in many neurodegenerative diseases, ensuring that we study synapses with accurate and reliable techniques is crucial. From high resolution imaging techniques such as array tomography, to highly sensitive molecular investigations using mass spectrometry, all have their advantages and limitations and the use of a particular tool in a study investigating synapses will depend on the study’s aims. Imaging technologies continue to improve and the study of protein changes at the synaptic scale are becoming ever more accessible. Furthermore, MS equipment is becoming ever more sensitive and sophisticated, providing deeper coverage and more accurate identification of protein content. Excitingly, these two seemingly disparate modalities are now beginning to overlap, and the development of spatial proteomics approaches may herald the future of synaptic analysis. By embracing new techniques and applying them to human disease, we may soon be able to understand the molecular changes driving synaptopathy and design new ways of stopping it, and in doing so, treat some of the most devastating diseases of our time.

## Author contributions

NH and AS wrote the manuscript and prepared the figures. CH helped to write the manuscript, plus edited, and formatted the final draft. All authors contributed to the article and approved the submitted version.

## References

[B1] Ahmad F. Liu P. (2020). Synaptosome as a tool in Alzheimer’s disease research. *Brain Res.* 1746:147009. 10.1016/j.brainres.2020.147009 32659233

[B2] Ardiles A. Grabrucker A. Scholl F. Rudenko G. Borsello T. (2017). Molecular and cellular mechanisms of synaptopathies. *Neural Plast.* 2017:2643943. 10.1155/2017/2643943 28540088 PMC5429942

[B3] Azevedo F. Carvalho L. Grinberg L. Farfel J. Ferretti R. Leite R. (2009). Equal numbers of neuronal and nonneuronal cells make the human brain an isometrically scaled-up primate brain. *J. Comp. Neurol.* 513 532–541. 10.1002/cne.21974 19226510

[B4] Bademosi A. Lauwers E. Amor R. Verstreken P. van Swinderen B. Meunier F. (2018). *In vivo* single-molecule tracking at the *Drosophila* presynaptic motor nerve terminal. *J. Vis. Exp.* 56952. 10.3791/56952 29364242 PMC5908646

[B5] Bellucci A. Longhena F. Spillantini M. G. (2022). The role of Rab proteins in Parkinson’s disease synaptopathy. *Biomedicines* 10:1941. 10.3390/biomedicines10081941 36009486 PMC9406004

[B6] Bereczki E. Branca R. Francis P. Pereira J. Baek J. Hortobágyi T. (2018). Synaptic markers of cognitive decline in neurodegenerative diseases: A proteomic approach. *Brain* 141 582–595. 10.1093/brain/awx352 29324989 PMC5837272

[B7] Bermejo M. Milenkovic M. Salahpour A. Ramsey A. (2014). Preparation of synaptic plasma membrane and postsynaptic density proteins using a discontinuous sucrose gradient. *J. Vis. Exp.* e51896. 10.3791/51896 25226023 PMC4828025

[B8] Betzig E. Patterson G. Sougrat R. Lindwasser O. Olenych S. Bonifacino J. (2006). Imaging intracellular fluorescent proteins at nanometer resolution. *Science* 313 1642–1645. 10.1126/science.1127344 16902090

[B9] Biesemann C. Grønborg M. Luquet E. Wichert S. Bernard V. Bungers S. (2014). Proteomic screening of glutamatergic mouse brain synaptosomes isolated by fluorescence activated sorting. *EMBO J.* 33 157–170. 10.1002/embj.201386120 24413018 PMC3989609

[B10] Bloss E. Cembrowski M. Karsh B. Colonell J. Fetter R. Spruston N. (2018). Single excitatory axons form clustered synapses onto CA1 pyramidal cell dendrites. *Nat. Neurosci.* 21 353–363. 10.1038/s41593-018-0084-6 29459763

[B11] Böger C. Hafner A.-S. Schlichthärle T. Strauss M. T. Malkusch S. Endesfelder U. (2019). Super-resolution imaging and estimation of protein copy numbers at single synapses with DNA-point accumulation for imaging in nanoscale topography. *Neurophotonics* 6:035008. 10.1117/1.NPh.6.3.035008 31637284 PMC6795074

[B12] Boyer D. Li B. Sun C. Fan W. Zhou K. Hughes M. (2020). The α-synuclein hereditary mutation E46K unlocks a more stable, pathogenic fibril structure. *Proc. Natl. Acad. Sci. U.S.A.* 117 3592–3602. 10.1073/pnas.1917914117 32015135 PMC7035510

[B13] Brinkmalm A. Brinkmalm G. Honer W. Moreno J. Jakobsson J. Mallucci G. (2014). Targeting synaptic pathology with a novel affinity mass spectrometry approach. *Mol. Cell Proteomics* 13 2584–2592. 10.1074/mcp.M114.040113 24973420 PMC4188988

[B14] Broadhead M. Horrocks M. Zhu F. Muresan L. Benavides-Piccione R. DeFelipe J. (2016). PSD95 nanoclusters are postsynaptic building blocks in hippocampus circuits. *Sci. Rep.* 6:24626. 10.1038/srep24626 27109929 PMC4842999

[B15] Cai Z. Li S. Matuskey D. Nabulsi N. Huang Y. (2019). PET imaging of synaptic density: A new tool for investigation of neuropsychiatric diseases. *Neurosci. Lett.* 691 44–50. 10.1016/j.neulet.2018.07.038 30075287 PMC6339829

[B16] Cao Q. Boyer D. Sawaya M. Ge P. Eisenberg D. (2019). Cryo-EM structures of four polymorphic TDP-43 amyloid cores. *Nat. Struct. Mol. Biol.* 26 619–627. 10.1038/s41594-019-0248-4 31235914 PMC7047951

[B17] Chen C. Hou J. Tanner J. Cheng J. (2020). Bioinformatics methods for mass spectrometry-based proteomics data analysis. *Int. J. Mol. Sci.* 21:2873. 10.3390/ijms21082873 32326049 PMC7216093

[B18] Chen F. Tillberg P. Boyden E. (2015). Optical imaging. Expansion microscopy. *Science* 347 543–548. 10.1126/science.1260088 25592419 PMC4312537

[B19] Chen M. Mecca A. Naganawa M. Finnema S. Toyonaga T. Lin S. (2018). Assessing synaptic density in Alzheimer disease with synaptic vesicle glycoprotein 2A positron emission tomographic imaging. *JAMA Neurol.* 75 1215–1224. 10.1001/jamaneurol.2018.1836 30014145 PMC6233853

[B20] Cho K. (2019). Emerging roles of complement protein C1q in neurodegeneration. *Aging Dis.* 10 652–663. 10.14336/AD.2019.0118 31165008 PMC6538225

[B21] Chung W.-S. Barres B. A. (2012). The role of glial cells in synapse elimination. *Curr. Opin. Neurobiol.* 22 438–445. 10.1016/j.conb.2011.10.003 22036016 PMC3319527

[B22] Collman F. Buchanan J. Phend K. Micheva K. Weinberg R. Smith S. (2015). Mapping synapses by conjugate light-electron array tomography. *J. Neurosci.* 35 5792–5807. 10.1523/JNEUROSCI.4274-14.2015 25855189 PMC4388933

[B23] Colom-Cadena M. Gelpi E. Charif S. Belbin O. Blesa R. Martí M. (2013). Confluence of α-synuclein, tau, and β-amyloid pathologies in dementia with Lewy bodies. *J. Neuropathol. Exp. Neurol.* 72 1203–1212. 10.1097/NEN.0000000000000018 24226269

[B24] Colom-Cadena M. Pegueroles J. Herrmann A. Henstridge C. Muñoz L. Querol-Vilaseca M. (2017). Synaptic phosphorylated α-synuclein in dementia with Lewy bodies. *Brain* 140 3204–3214. 10.1093/brain/awx275 29177427 PMC5841145

[B25] Crosby K. Gookin S. Garcia J. Hahm K. Dell’Acqua M. Smith K. (2019). Nanoscale subsynaptic domains underlie the organization of the inhibitory synapse. *Cell Rep.* 26 3284–3297.e3. 10.1016/j.celrep.2019.02.070 30893601 PMC6529211

[B26] Crowther R. Daniel S. Goedert M. (2000). Characterisation of isolated alpha-synuclein filaments from substantia nigra of Parkinson’s disease brain. *Neurosci. Lett.* 292 128–130. 10.1016/s0304-3940(00)01440-3 10998565

[B27] Dalakas M. Alexopoulos H. Spaeth P. (2020). Complement in neurological disorders and emerging complement-targeted therapeutics. *Nat. Rev. Neurol.* 16 601–617. 10.1038/s41582-020-0400-0 33005040 PMC7528717

[B28] DeKosky S. Scheff S. (1990). Synapse loss in frontal cortex biopsies in Alzheimer’s disease: Correlation with cognitive severity. *Ann. Neurol.* 27 457–464. 10.1002/ana.410270502 2360787

[B29] Deshpande D. Higelin J. Schoen M. Vomhof T. Boeckers T. Demestre M. (2019). Synaptic FUS localization during motoneuron development and its accumulation in human ALS synapses. *Front. Cell Neurosci.* 13:256. 10.3389/fncel.2019.00256 31244613 PMC6582137

[B30] di Benedetto G. Burgaletto C. Bellanca C. M. Munafò A. Bernardini R. Cantarella G. (2022). Role of microglia and astrocytes in Alzheimer’s disease: From neuroinflammation to Ca2+ homeostasis dysregulation. *Cells* 11:2728. 10.3390/cells11172728 36078138 PMC9454513

[B31] Dityatev A. Rusakov D. A. (2011). Molecular signals of plasticity at the tetrapartite synapse. *Curr. Opin. Neurobiol.* 21 353–359. 10.1016/j.conb.2010.12.006 21277196 PMC3368316

[B32] Eisenstein M. (2015). Super-resolve me: From micro to nano. *Nature* 526 459–462. 10.1038/526459a 26469055

[B33] Fatoba O. Itokazu T. Yamashita T. (2022). Complement cascade functions during brain development and neurodegeneration. *FEBS J.* 289 2085–2109. 10.1111/febs.15772 33599083

[B34] Finnema S. Nabulsi N. Eid T. Detyniecki K. Lin S. Chen M. (2016). Imaging synaptic density in the living human brain. *Sci. Transl. Med.* 8:348ra96. 10.1126/scitranslmed.aaf6667 27440727

[B35] Fitzpatrick A. Falcon B. He S. Murzin A. Murshudov G. Garringer H. (2017). Cryo-EM structures of tau filaments from Alzheimer’s disease. *Nature* 547 185–190. 10.1038/nature23002 28678775 PMC5552202

[B36] Fuhrmann M. Gockel N. Arizono M. Dembitskaya Y. Nägerl U. Pennacchietti F. (2022). Super-resolution microscopy opens new doors to life at the nanoscale. *J. Neurosci.* 42 8488–8497. 10.1523/JNEUROSCI.1125-22.2022 36351828 PMC9665916

[B37] Gajera C. R. Fernandez R. Postupna N. Montine K. S. Fox E. J. Tebaykin D. (2019). Mass synaptometry: High-dimensional multi parametric assay for single synapses. *J. Neurosci. Methods* 312 73–83. 10.1016/j.jneumeth.2018.11.008 30465796 PMC6363340

[B38] Gallagher B. Zhao Y. (2021). Expansion microscopy: A powerful nanoscale imaging tool for neuroscientists. *Neurobiol. Dis.* 154:105362. 10.1016/j.nbd.2021.105362 33813047 PMC8600979

[B39] Ge S. Jung D. Yao R. (2020). ShinyGO: A graphical gene-set enrichment tool for animals and plants. *Bioinformatics* 36 2628–2629. 10.1093/bioinformatics/btz931 31882993 PMC7178415

[B40] Gillespie M. Jassal B. Stephan R. Milacic M. Rothfels K. Senff-Ribeiro A. (2022). The reactome pathway knowledgebase 2022. *Nucleic Acids Res.* 50 D687–D692. 10.1093/nar/gkab1028 34788843 PMC8689983

[B41] Gomez-Arboledas A. Acharya M. Tenner A. (2021). The role of complement in synaptic pruning and neurodegeneration. *Immunotargets Ther.* 10 373–386. 10.2147/ITT.S305420 34595138 PMC8478425

[B42] Gopalakrishnan G. Yam P. Madwar C. Bostina M. Rouiller I. Colman D. (2011). Label-free visualization of ultrastructural features of artificial synapses via cryo-EM. *ACS Chem. Neurosci.* 2 700–704. 10.1021/cn200094j 22860164 PMC3369721

[B43] Groemer T. Thiel C. Holt M. Riedel D. Hua Y. Hüve J. (2011). Amyloid precursor protein is trafficked and secreted via synaptic vesicles. *PLoS One* 6:e18754. 10.1371/journal.pone.0018754 21556148 PMC3083403

[B44] Guerrero-Ferreira R. Taylor N. Arteni A. Kumari P. Mona D. Ringler P. (2019). Two new polymorphic structures of human full-length alpha-synuclein fibrils solved by cryo-electron microscopy. *Elife* 8:e48907. 10.7554/eLife.48907 31815671 PMC6957273

[B45] Gulyássy P. Puska G. Györffy B. A. Todorov-Völgyi K. Juhász G. Drahos L. (2020). Proteomic comparison of different synaptosome preparation procedures. *Amino Acids* 52 1529–1543. 10.1007/s00726-020-02912-6 33211194 PMC7695668

[B46] Guo Q. Lehmer C. Martínez-Sánchez A. Rudack T. Beck F. Hartmann H. (2018). In situ structure of neuronal C9orf72 Poly-GA aggregates reveals proteasome recruitment. *Cell* 172 696–705.e12. 10.1016/j.cell.2017.12.030 29398115 PMC6035389

[B47] Gustafsson M. (2000). Surpassing the lateral resolution limit by a factor of two using structured illumination microscopy. *J. Microsc.* 198 82–87. 10.1046/j.1365-2818.2000.00710.x 10810003

[B48] Hafner A. Donlin-Asp P. Leitch B. Herzog E. Schuman E. (2019). Local protein synthesis is a ubiquitous feature of neuronal pre- and postsynaptic compartments. *Science* 364:eaau3644. 10.1126/science.aau3644 31097639

[B49] Hasin Y. Seldin M. Lusis A. (2017). Multi-omics approaches to disease. *Genome Biol.* 18:83. 10.1186/s13059-017-1215-1 28476144 PMC5418815

[B50] Hell S. Wichmann J. (1994). Breaking the diffraction resolution limit by stimulated emission: Stimulated-emission-depletion fluorescence microscopy. *Opt. Lett.* 19 780–782. 10.1364/ol.19.000780 19844443

[B51] Henstridge C. M. Tzioras M. Paolicelli R. C. (2019). Glial contribution to excitatory and inhibitory synapse loss in neurodegeneration. *Front. Cell. Neurosci.* 13:63. 10.3389/fncel.2019.00063 30863284 PMC6399113

[B52] Henstridge C. Sideris D. Carroll E. Rotariu S. Salomonsson S. Tzioras M. (2018). Synapse loss in the prefrontal cortex is associated with cognitive decline in amyotrophic lateral sclerosis. *Acta Neuropathol.* 135 213–226. 10.1007/s00401-017-1797-4 29273900 PMC5773656

[B53] Hesse R. Hurtado M. Jackson R. Eaton S. Herrmann A. Colom-Cadena M. (2019). Comparative profiling of the synaptic proteome from Alzheimer’s disease patients with focus on the APOE genotype. *Acta Neuropathol. Commun.* 7:214. 10.1186/s40478-019-0847-7 31862015 PMC6925519

[B54] Hobson B. Choi S. Mosharov E. Soni R. Sulzer D. Sims P. (2022). Subcellular proteomics of dopamine neurons in the mouse brain. *Elife* 11:e70921. 10.7554/eLife.70921 35098924 PMC8860448

[B55] Holderith N. Heredi J. Kis V. Nusser Z. (2020). A high-resolution method for quantitative molecular analysis of functionally characterized individual synapses. *Cell Rep.* 32:107968. 10.1016/j.celrep.2020.107968 32726631 PMC7408500

[B56] Holland N. Malpetti M. Rittman T. Mak E. Passamonti L. Kaalund S. (2022). Molecular pathology and synaptic loss in primary tauopathies: An 18F-AV-1451 and 11C-UCB-J PET study. *Brain* 145 340–348. 10.1093/brain/awab282 34398211 PMC8967099

[B57] Hollingsworth E. McNeal E. Burton J. Williams R. Daly J. Creveling C. (1985). Biochemical characterization of a filtered synaptoneurosome preparation from guinea pig cerebral cortex: Cyclic adenosine 3’:5’-monophosphate-generating systems, receptors, and enzymes. *J. Neurosci.* 5 2240–2253. 10.1523/JNEUROSCI.05-08-02240.1985 2991484 PMC6565304

[B58] Hong S. Beja-Glasser V. Nfonoyim B. Frouin A. Li S. Ramakrishnan S. (2016). Complement and microglia mediate early synapse loss in Alzheimer mouse models. *Science* 352 712–716. 10.1126/science.aad8373 27033548 PMC5094372

[B59] Johnson M. Chotiner J. Watson J. (1997). Isolation and characterization of synaptoneurosomes from single rat hippocampal slices. *J. Neurosci. Methods* 77 151–156. 10.1016/s0165-0270(97)00120-9 9489891

[B60] Jones R. Harrison C. Eaton S. Llavero Hurtado M. Graham L. Alkhammash L. (2017). Cellular and molecular anatomy of the human neuromuscular junction. *Cell Rep.* 21 2348–2356. 10.1016/j.celrep.2017.11.008 29186674 PMC5723673

[B61] Jung K. Sepers M. Henstridge C. Lassalle O. Neuhofer D. Martin H. (2012). Uncoupling of the endocannabinoid signalling complex in a mouse model of fragile X syndrome. *Nat. Commun.* 3:1080. 10.1038/ncomms2045 23011134 PMC3657999

[B62] Kanehisa M. Goto S. (2000). KEGG: Kyoto encyclopedia of genes and genomes. *Nucleic Acids Res.* 28 27–30. 10.1093/nar/28.1.27 10592173 PMC102409

[B63] Katsuse O. Lin W. Lewis J. Hutton M. Dickson D. (2006). Neurofibrillary tangle-related synaptic alterations of spinal motor neurons of P301L tau transgenic mice. *Neurosci. Lett.* 409 95–99. 10.1016/j.neulet.2006.09.021 17010516

[B64] Kay K. Smith C. Wright A. Serrano-Pozo A. Pooler A. Koffie R. (2013). Studying synapses in human brain with array tomography and electron microscopy. *Nat. Protoc.* 8 1366–1380. 10.1038/nprot.2013.078 23787894 PMC3712649

[B65] Kerrigan T. L. Randall A. D. (2013). A new player in the “synaptopathy” of Alzheimer’s disease – Arc/Arg 3.1. *Front. Neurol.* 4:9. 10.3389/fneur.2013.00009 23407382 PMC3570765

[B66] Kim G. Bahn S. Kim N. Choi J. Kim J. Rah J. (2021). Efficient and accurate synapse detection with selective structured illumination microscopy on the putative regions of interest of ultrathin serial sections. *Front. Neuroanat.* 15:759816. 10.3389/fnana.2021.759816 34867216 PMC8634652

[B67] Kim Y. S. Choi J. Yoon B.-E. (2020). Neuron-glia interactions in neurodevelopmental disorders. *Cells* 9:2176. 10.3390/cells9102176 32992620 PMC7601502

[B68] Kleinfeld D. Bharioke A. Blinder P. Bock D. Briggman K. Chklovskii D. (2011). Large-scale automated histology in the pursuit of connectomes. *J. Neurosci.* 31 16125–16138. 10.1523/JNEUROSCI.4077-11.2011 22072665 PMC3758571

[B69] Kline R. Lößlein L. Kurian D. Aguilar Martí J. Eaton S. Court F. (2022). An optimized comparative proteomic approach as a tool in neurodegenerative disease research. *Cells* 11:2653. 10.3390/cells11172653 36078061 PMC9454658

[B70] Koffie R. Hashimoto T. Tai H. Kay K. Serrano-Pozo A. Joyner D. (2012). Apolipoprotein E4 effects in Alzheimer’s disease are mediated by synaptotoxic oligomeric amyloid-β. *Brain.* 135 2155–2168. 10.1093/brain/aws127 22637583 PMC3381721

[B71] Koffie R. Meyer-Luehmann M. Hashimoto T. Adams K. Mielke M. Garcia-Alloza M. (2009). Oligomeric amyloid beta associates with postsynaptic densities and correlates with excitatory synapse loss near senile plaques. *Proc. Natl. Acad. Sci. U.S.A.* 106 4012–4017. 10.1073/pnas.0811698106 19228947 PMC2656196

[B72] Kono R. Ikegaya Y. Koyama R. (2021). Phagocytic glial cells in brain homeostasis. *Cells* 10:1348. 10.3390/cells10061348 34072424 PMC8229427

[B73] Koopmans F. van Nierop P. Andres-Alonso M. Byrnes A. Cijsouw T. Coba M. (2019). SynGO: An evidence-based, expert-curated knowledge base for the synapse. *Neuron* 103 217–234.e4. 10.1016/j.neuron.2019.05.002 31171447 PMC6764089

[B74] Krämer A. Green J. Pollard J. Tugendreich S. (2014). Causal analysis approaches in ingenuity pathway analysis. *Bioinformatics* 30 523–530. 10.1093/bioinformatics/btt703 24336805 PMC3928520

[B75] Krasny L. Huang P. (2021). Data-independent acquisition mass spectrometry (DIA-MS) for proteomic applications in oncology. *Mol. Omics* 17 29–42. 10.1039/d0mo00072h 33034323

[B76] Kumar S. Orlov E. Gowda P. Bose C. Swerdlow R. Lahiri D. (2022). Synaptosome microRNAs regulate synapse functions in Alzheimer’s disease. *NPJ Genom. Med.* 7:47. 10.1038/s41525-022-00319-8 35941185 PMC9359989

[B77] LaCava J. Molloy K. Taylor M. Domanski M. Chait B. Rout M. (2015). Affinity proteomics to study endogenous protein complexes: Pointers, pitfalls, preferences and perspectives. *Biotechniques* 58 103–119. 10.2144/000114262 25757543 PMC4465938

[B78] Lambert T. Waters J. (2017). Navigating challenges in the application of superresolution microscopy. *J. Cell Biol.* 216 53–63. 10.1083/jcb.201610011 27920217 PMC5223610

[B79] Laszlo Z. Hindley N. Sanchez Avila A. Kline R. Eaton S. Lamont D. (2022). Synaptic proteomics reveal distinct molecular signatures of cognitive change and C9ORF72 repeat expansion in the human ALS cortex. *Acta Neuropathol. Commun.* 10:156. 10.1186/s40478-022-01455-z 36309735 PMC9618182

[B80] Lim J. Yue Z. (2015). Neuronal aggregates: Formation, clearance, and spreading. *Dev. Cell* 32 491–501. 10.1016/j.devcel.2015.02.002 25710535 PMC4376477

[B81] Liu Y. Shen X. Zhang Y. Zheng X. Cepeda C. Wang Y. (2023). Interactions of glial cells with neuronal synapses, from astrocytes to microglia and oligodendrocyte lineage cells. *Glia.* 1–19. 10.1002/glia.24343 36799296

[B82] Liu Y. Tao C. Zhang X. Xia W. Shi D. Qi L. (2020). Mesophasic organization of GABAA receptors in hippocampal inhibitory synapses. *Nat. Neurosci.* 23 1589–1596. 10.1038/s41593-020-00729-w 33139942 PMC8048127

[B83] Mallozzi C. Spalloni A. Longone P. Domenici M. (2018). Activation of phosphotyrosine-mediated signaling pathways in the cortex and spinal cord of SOD1G93A, a mouse model of familial amyotrophic lateral sclerosis. *Neural Plast.* 2018:2430193. 10.1155/2018/2430193 30154836 PMC6098854

[B84] Malpetti M. Holland N. Jones P. Ye R. Cope T. Fryer T. (2021). Synaptic density in carriers of C9orf72 mutations: A [11 C]UCB-J PET study. *Ann. Clin. Transl. Neurol.* 8 1515–1523. 10.1002/acn3.51407 34133849 PMC8283163

[B85] Malpetti M. Jones P. Cope T. Holland N. Naessens M. Rouse M. (2023). Synaptic loss in frontotemporal dementia revealed by [11 C]UCB-J positron emission tomography. *Ann. Neurol.* 93 142–154. 10.1002/ana.26543 36321699 PMC10099663

[B86] Marcassa G. Dascenco D. de Wit J. (2023). Proteomics-based synapse characterization: From proteins to circuits. *Curr. Opin. Neurobiol.* 79:102690. 10.1016/j.conb.2023.102690 36805717

[B87] Masch J.-M. Steffens H. Fischer J. Engelhardt J. Hubrich J. Keller-Findeisen J. (2018). Robust nanoscopy of a synaptic protein in living mice by organic-fluorophore labeling. *Proc. Natl. Acad. Sci. U.S.A.* 115, E8047–E8056. 10.1073/pnas.1807104115 30082388 PMC6112726

[B88] Matsubara A. Laake J. Davanger S. Usami S. Ottersen O. (1996). Organization of AMPA receptor subunits at a glutamate synapse: A quantitative immunogold analysis of hair cell synapses in the rat organ of Corti. *J. Neurosci.* 16 4457–4467. 10.1523/JNEUROSCI.16-14-04457.1996 8699256 PMC6578857

[B89] Matuskey D. Tinaz S. Wilcox K. Naganawa M. Toyonaga T. Dias M. (2020). Synaptic changes in Parkinson disease assessed with *in vivo* imaging. *Ann. Neurol.* 87 329–338. 10.1002/ana.25682 31953875 PMC7065227

[B90] Mecca A. Chen M. O’Dell R. Naganawa M. Toyonaga T. Godek T. (2020). *In vivo* measurement of widespread synaptic loss in Alzheimer’s disease with SV2A PET. *Alzheimers Dement.* 16 974–982. 10.1002/alz.12097 32400950 PMC7383876

[B91] Micheva K. Smith S. (2007). Array tomography: A new tool for imaging the molecular architecture and ultrastructure of neural circuits. *Neuron* 55 25–36. 10.1016/j.neuron.2007.06.014 17610815 PMC2080672

[B92] Micheva K. Kiraly M. Perez M. Madison D. (2021). Conduction velocity along the local axons of parvalbumin interneurons correlates with the degree of axonal myelination. *Cereb. Cortex* 31 3374–3392. 10.1093/cercor/bhab018 33704414 PMC8196249

[B93] Morizawa Y. Matsumoto M. Nakashima Y. Endo N. Aida T. Ishikane H. (2022). Synaptic pruning through glial synapse engulfment upon motor learning. *Nat. Neurosci.* 25 1458–1469. 10.1038/s41593-022-01184-5 36319770

[B94] Murphy-Royal C. Dupuis J. Groc L. Oliet S. H. R. (2017). Astroglial glutamate transporters in the brain: Regulating neurotransmitter homeostasis and synaptic transmission. *J. Neurosci. Res.* 95 2140–2151. 10.1002/jnr.24029 28150867

[B95] Nishimura A. L. Arias N. (2021). Synaptopathy mechanisms in ALS caused by C9orf72 repeat expansion. *Front. Cell. Neurosci.* 15:660693. 10.3389/fncel.2021.660693 34140881 PMC8203826

[B96] Nowicka M. Krieg C. Crowell H. Weber L. Hartmann F. Guglietta S. (2017). CyTOF workflow: Differential discovery in high-throughput high-dimensional cytometry datasets. *F1000Research* 6:748. 10.12688/f1000research.11622.3 28663787 PMC5473464

[B97] Nusser Z. Mulvihill E. Streit P. Somogyi P. (1994). Subsynaptic segregation of metabotropic and ionotropic glutamate receptors as revealed by immunogold localization. *Neuroscience* 61 421–427. 10.1016/0306-4522(94)90421-9 7969918

[B98] O’Dell R. Mecca A. Chen M. Naganawa M. Toyonaga T. Lu Y. (2021). Association of Aβ deposition and regional synaptic density in early Alzheimer’s disease: A PET imaging study with [11C]UCB-J. *Alzheimers Res. Ther.* 13:11. 10.1186/s13195-020-00742-y 33402201 PMC7786921

[B99] Obi-Nagata K. Temma Y. Hayashi-Takagi A. (2019). Synaptic functions and their disruption in schizophrenia: From clinical evidence to synaptic optogenetics in an animal model. *Proc. Jpn. Acad. Ser. B Phys. Biol. Sci.* 95 179–197. 10.2183/pjab.95.014 31080187 PMC6742729

[B100] Padmanabhan P. Kneynsberg A. Götz J. (2021). Super-resolution microscopy: A closer look at synaptic dysfunction in Alzheimer disease. *Nat. Rev. Neurosci.* 22 723–740. 10.1038/s41583-021-00531-y 34725519

[B101] Paik S. Yoshida A. Bae Y. (2021). Development of γ-aminobutyric acid-, glycine-, and glutamate-immunopositive boutons on the rat genioglossal motoneurons. *Brain Struct. Funct.* 226 889–900. 10.1007/s00429-021-02216-9 33475854

[B102] Perea G. Navarrete M. Araque A. (2009). Tripartite synapses: Astrocytes process and control synaptic information. *Trends Neurosci.* 32 421–431. 10.1016/j.tins.2009.05.001 19615761

[B103] Petralia R. Wang Y. (2021). Review of post-embedding immunogold methods for the study of neuronal structures. *Front. Neuroanat.* 15:763427. 10.3389/fnana.2021.763427 34720893 PMC8551803

[B104] Petralia R. Wang Y. Sans N. Worley P. Hammer J. Wenthold R. (2001). Glutamate receptor targeting in the postsynaptic spine involves mechanisms that are independent of myosin Va. *Eur. J. Neurosci.* 13 1722–1732. 10.1046/j.0953-816x.2001.01553.x 11359524

[B105] Petralia R. Zhao H. Wang Y. Wenthold R. (1998). Variations in the tangential distribution of postsynaptic glutamate receptors in Purkinje cell parallel and climbing fiber synapses during development. *Neuropharmacology* 37 1321–1334. 10.1016/s0028-3908(98)00118-x 9849668

[B106] Petrov K. A. Proskurina S. E. Krejci E. (2021). Cholinesterases in tripartite neuromuscular synapse. *Front. Mol. Neurosci.* 14:811220. 10.3389/fnmol.2021.811220 35002624 PMC8733319

[B107] Phend K. Weinberg R. Rustioni A. (1992). Techniques to optimize post-embedding single and double staining for amino acid neurotransmitters. *J. Histochem. Cytochem.* 40 1011–1020. 10.1177/40.7.1376741 1376741

[B108] Pickett E. Herrmann A. McQueen J. Abt K. Dando O. Tulloch J. (2019). Amyloid beta and tau cooperate to cause reversible behavioral and transcriptional deficits in a model of Alzheimer’s disease. *Cell Rep.* 29 3592–3604.e5. 10.1016/j.celrep.2019.11.044 31825838 PMC6915767

[B109] Pickett E. Koffie R. Wegmann S. Henstridge C. Herrmann A. Colom-Cadena M. (2016). Non-fibrillar oligomeric amyloid-β within synapses. *J. Alzheimers Dis.* 53 787–800. 10.3233/JAD-160007 27258414

[B110] Pickett E. Rose J. McCrory C. McKenzie C. King D. Smith C. (2018). Region-specific depletion of synaptic mitochondria in the brains of patients with Alzheimer’s disease. *Acta Neuropathol.* 136 747–757. 10.1007/s00401-018-1903-2 30191401 PMC6208730

[B111] Plum S. Eggers B. Helling S. Stepath M. Theiss C. Leite R. (2020). Proteomic characterization of synaptosomes from human substantia nigra indicates altered mitochondrial translation in Parkinson’s disease. *Cells* 9:2580. 10.3390/cells9122580 33276480 PMC7761546

[B112] Qiu H. Lee S. Shang Y. Wang W. Au K. Kamiya S. (2014). ALS-associated mutation FUS-R521C causes DNA damage and RNA splicing defects. *J. Clin. Invest.* 124 981–999. 10.1172/JCI72723 24509083 PMC3938263

[B113] Querol-Vilaseca M. Colom-Cadena M. Pegueroles J. Nuñez-Llaves R. Luque-Cabecerans J. Muñoz-Llahuna L. (2019). Nanoscale structure of amyloid-β plaques in Alzheimer’s disease. *Sci. Rep.* 9:5181. 10.1038/s41598-019-41443-3 30914681 PMC6435662

[B114] Raduloviæ S. Sunkara S. Maurer C. Leitinger G. (2021). Digging deeper: Advancements in visualization of inhibitory synapses in neurodegenerative disorders. *Int. J. Mol. Sci.* 22:12470. 10.3390/ijms222212470 34830352 PMC8623765

[B115] Rajkumar S. Böckers T. M. Catanese A. (2023). Fast and efficient synaptosome isolation and post-synaptic density enrichment from hiPSC-motor neurons by biochemical sub-cellular fractionation. *STAR Protoc.* 4:102061. 10.1016/j.xpro.2023.102061 36853677 PMC9898788

[B116] Raudvere U. Kolberg L. Kuzmin I. Arak T. Adler P. Peterson H. (2019). g:Profiler: A web server for functional enrichment analysis and conversions of gene lists (2019 update). *Nucleic Acids Res.* 47 W191–W198. 10.1093/nar/gkz369 31066453 PMC6602461

[B117] Robbins M. Clayton E. Kaminski Schierle G. S. (2021). Synaptic tau: A pathological or physiological phenomenon? *Acta Neuropathol. Commun.* 9:149. 10.1186/s40478-021-01246-y 34503576 PMC8428049

[B118] Rossano S. Toyonaga T. Bini J. Nabulsi N. Ropchan J. Cai Z. (2022). Feasibility of imaging synaptic density in the human spinal cord using [11C]UCB-J PET. *EJNMMI Phys.* 9:32. 10.1186/s40658-022-00464-0 35503134 PMC9065222

[B119] Rupawala H. Shah K. Davies C. Rose J. Colom-Cadena M. Peng X. (2022). Cysteine string protein alpha accumulates with early pre-synaptic dysfunction in Alzheimer’s disease. *Brain Commun.* 4:fcac192. 10.1093/braincomms/fcac192 35928052 PMC9345313

[B120] Rust M. Bates M. Zhuang X. (2006). Sub-diffraction-limit imaging by stochastic optical reconstruction microscopy (STORM). *Nat Methods.* 3 793–795. 10.1038/nmeth929 16896339 PMC2700296

[B121] Sanchez Avila A. Henstridge C. (2022). Array tomography: 15 years of synaptic analysis. *Neuronal. Signal.* 6:NS20220013. 10.1042/NS20220013 36187224 PMC9512143

[B122] Sapp E. Seeley C. Iuliano M. Weisman E. Vodicka P. DiFiglia M. (2020). Protein changes in synaptosomes of Huntington’s disease knock-in mice are dependent on age and brain region. *Neurobiol. Dis.* 141:104950. 10.1016/j.nbd.2020.104950 32439598

[B123] Sarkar D. Kang J. Wassie A. Schroeder M. Peng Z. Tarr T. (2022). Revealing nanostructures in brain tissue via protein decrowding by iterative expansion microscopy. *Nat. Biomed. Eng.* 6 1057–1073. 10.1038/s41551-022-00912-3 36038771 PMC9551354

[B124] Sasaki S. Iwata M. (1996a). Ultrastructural study of the synapses of central chromatolytic anterior horn cells in motor neuron disease. *J. Neuropathol. Exp. Neurol.* 55 932–939. 10.1097/00005072-199608000-00009 8759783

[B125] Sasaki S. Iwata M. (1996b). Ultrastructural study of synapses in the anterior horn neurons of patients with amyotrophic lateral sclerosis. *Neurosci. Lett.* 204 53–56. 10.1016/0304-3940(96)12314-4 8929976

[B126] Sasaki S. Maruyama S. (1994a). Synapse loss in anterior horn neurons in amyotrophic lateral sclerosis. *Acta Neuropathol.* 88 222–227. 10.1007/BF00293397 7810293

[B127] Sasaki S. Maruyama S. (1994b). Decreased synaptophysin immunoreactivity of the anterior horns in motor neuron disease. *Acta Neuropathol.* 87 125–128. 10.1007/BF00296180 8171961

[B128] Sauerbeck A. Gangolli M. Reitz S. Salyards M. Kim S. Hemingway C. (2020). SEQUIN multiscale imaging of mammalian central synapses reveals loss of synaptic connectivity resulting from diffuse traumatic brain injury. *Neuron* 107 257–273.e5. 10.1016/j.neuron.2020.04.012 32392471 PMC7381374

[B129] Schedin-Weiss S. Caesar I. Winblad B. Blom H. Tjernberg L. (2016). Super-resolution microscopy reveals γ-secretase at both sides of the neuronal synapse. *Acta Neuropathol. Commun.* 4:29. 10.1186/s40478-016-0296-5 27036709 PMC4818506

[B130] Scheff S. Sparks L. Price D. (1993). Quantitative assessment of synaptic density in the entorhinal cortex in Alzheimer’s disease. *Ann. Neurol.* 34 356–361. 10.1002/ana.410340309 8363352

[B131] Shahidullah M. Le Marchand S. Fei H. Zhang J. Pandey U. Dalva M. (2013). Defects in synapse structure and function precede motor neuron degeneration in *Drosophila* models of FUS-related ALS. *J. Neurosci.* 33 19590–19598. 10.1523/JNEUROSCI.3396-13.2013 24336723 PMC3858628

[B132] Shan L. Zhang T. Fan K. Cai W. Liu H. (2021). Astrocyte-neuron signaling in synaptogenesis. *Front. Cell Dev. Biol.* 9:680301. 10.3389/fcell.2021.680301 34277621 PMC8284252

[B133] Sherman B. Hao M. Qiu J. Jiao X. Baseler M. Lane H. (2022). DAVID: A web server for functional enrichment analysis and functional annotation of gene lists (2021 update). *Nucleic Acids Res.* 50 W216–W221. 10.1093/nar/gkac194 35325185 PMC9252805

[B134] Sigrist S. Sabatini B. (2012). Optical super-resolution microscopy in neurobiology. *Curr. Opin. Neurobiol.* 22 86–93. 10.1016/j.conb.2011.10.014 22051692

[B135] Spillantini M. Schmidt M. Lee V. Trojanowski J. Jakes R. Goedert M. (1997). Alpha-synuclein in Lewy bodies. *Nature* 388 839–840. 10.1038/42166 9278044

[B136] Spires T. Meyer-Luehmann M. Stern E. McLean P. Skoch J. Nguyen P. (2005). Dendritic spine abnormalities in amyloid precursor protein transgenic mice demonstrated by gene transfer and intravital multiphoton microscopy. *J. Neurosci.* 25 7278–7287. 10.1523/JNEUROSCI.1879-05.2005 16079410 PMC1820616

[B137] Spires-Jones T. Hyman B. (2014). The intersection of amyloid beta and tau at synapses in Alzheimer’s disease. *Neuron* 82 756–771. 10.1016/j.neuron.2014.05.004 24853936 PMC4135182

[B138] Strack R. (2018). Gentler super-resolution microscopy. *Nat. Methods* 15:764. 10.1038/s41592-018-0159-z 30275581

[B139] Suzuki K. Elegheert J. Song I. Sasakura H. Senkov O. Matsuda K. (2020). A synthetic synaptic organizer protein restores glutamatergic neuronal circuits. *Science* 369:eabb4853. 10.1126/science.abb4853 32855309 PMC7116145

[B140] Tai H. Serrano-Pozo A. Hashimoto T. Frosch M. Spires-Jones T. Hyman B. (2012). The synaptic accumulation of hyperphosphorylated tau oligomers in Alzheimer disease is associated with dysfunction of the ubiquitin-proteasome system. *Am. J. Pathol.* 181 1426–1435. 10.1016/j.ajpath.2012.06.033 22867711 PMC3463637

[B141] Takano T. Soderling S. H. (2021). Tripartite synaptomics: Cell-surface proximity labeling *in vivo*. *Neurosci. Res.* 173 14–21. 10.1016/j.neures.2021.05.002 34019951 PMC8602446

[B142] Tang Y. Liu P. Li W. Liu Z. Zhou M. Li J. (2022). Detection of changes in synaptic density in amyotrophic lateral sclerosis patients using 18 F-SynVesT-1 positron emission tomography. *Eur. J. Neurol.* 29 2934–2943. 10.1111/ene.15451 35708508

[B143] Tao-Cheng J. Crocker V. Moreira S. Azzam R. (2021). Optimization of protocols for pre-embedding immunogold electron microscopy of neurons in cell cultures and brains. *Mol. Brain* 14:86. 10.1186/s13041-021-00799-2 34082785 PMC8173732

[B144] Taoufik E. Kouroupi G. Zygogianni O. Matsas R. (2018). Synaptic dysfunction in neurodegenerative and neurodevelopmental diseases: An overview of induced pluripotent stem-cell-based disease models. *Open Biol.* 8:180138. 10.1098/rsob.180138 30185603 PMC6170506

[B145] Timp W. Timp G. (2020). Beyond mass spectrometry, the next step in proteomics. *Sci. Adv.* 6:eaax8978. 10.1126/sciadv.aax8978 31950079 PMC6954058

[B146] Titze B. Genoud C. (2016). Volume scanning electron microscopy for imaging biological ultrastructure. *Biol. Cell* 108 307–323. 10.1111/boc.201600024 27432264

[B147] Tønnesen J. Nägerl U. (2013). Superresolution imaging for neuroscience. *Exp. Neurol.* 242 33–40. 10.1016/j.expneurol.2012.10.004 23063602

[B148] Torres V. Vallejo D. Inestrosa N. (2017). Emerging synaptic molecules as candidates in the etiology of neurological disorders. *Neural Plast.* 2017:8081758. 10.1155/2017/8081758 28331639 PMC5346360

[B149] Um J. (2017). Roles of glial cells in sculpting inhibitory synapses and neural circuits. *Front. Mol. Neurosci.* 10:381. 10.3389/fnmol.2017.00381 29180953 PMC5694142

[B150] Umoh M. Dammer E. Dai J. Duong D. Lah J. Levey A. (2018). A proteomic network approach across the ALS-FTD disease spectrum resolves clinical phenotypes and genetic vulnerability in human brain. *EMBO Mol. Med.* 10 48–62. 10.15252/emmm.201708202 29191947 PMC5760858

[B151] Valenzuela R. Micheva K. Kiraly M. Li D. Madison D. (2016). Array tomography of physiologically-characterized CNS synapses. *J. Neurosci. Methods* 268 43–52. 10.1016/j.jneumeth.2016.04.017 27141856 PMC5501355

[B152] Valtschanoff J. Weinberg R. (2001). Laminar organization of the NMDA receptor complex within the postsynaptic density. *J. Neurosci.* 21 1211–1217. 10.1523/JNEUROSCI.21-04-01211.2001 11160391 PMC6762240

[B153] van Oostrum M. Blok T. Giandomenico S. tom Dieck S. Tushev G. Nicole Fürst N. (2023). The proteomic landscape of synaptic diversity across brain regions and cell types. *bioRxiv* [Preprint]. 10.1101/2023.01.27.525780 PMC10686415 37918396

[B154] Velásquez E. Nogueira F. C. S. Velásquez I. Schmitt A. Falkai P. Domont G. B. (2017). Synaptosomal proteome of the orbitofrontal cortex from schizophrenia patients using quantitative label-free and iTRAQ-based shotgun proteomics. *J. Proteome Res.* 16 4481–4494. 10.1021/acs.jproteome.7b00422 28949146

[B155] Wang Y.-C. Lauwers E. Verstreken P. (2017). Presynaptic protein homeostasis and neuronal function. *Curr. Opin. Genet. Dev.* 44 38–46. 10.1016/j.gde.2017.01.015 28213157

[B156] Weiler I. J. (2009). “Synaptosomes,” in *Encyclopedia of neuroscience*, ed. L. R. Squire (Amsterdam: Elsevier), 815–818. 10.1016/B978-008045046-9.02045-3

[B157] Whittaker V. Michaelson I. Kirkland R. (1964). The separation of synaptic vesicles from nerve-ending particles (‘synaptosomes’). *Biochem. J.* 90 293–303. 10.1042/bj0900293 5834239 PMC1202615

[B158] Wijasa T. Sylvester M. Brocke-Ahmadinejad N. Schwartz S. Santarelli F. Gieselmann V. (2020). Quantitative proteomics of synaptosome S-nitrosylation in Alzheimer’s disease. *J. Neurochem.* 152 710–726. 10.1111/jnc.14870 31520481

[B159] Wu H. Hudry E. Hashimoto T. Kuchibhotla K. Rozkalne A. Fan Z. (2010). Amyloid beta induces the morphological neurodegenerative triad of spine loss, dendritic simplification, and neuritic dystrophies through calcineurin activation. *J. Neurosci.* 30 2636–2649. 10.1523/JNEUROSCI.4456-09.2010 20164348 PMC2841957

[B160] Xiao B. Tu J. Petralia R. Yuan J. Doan A. Breder C. (1998). Homer regulates the association of group 1 metabotropic glutamate receptors with multivalent complexes of homer-related, synaptic proteins. *Neuron* 21 707–716. 10.1016/s0896-6273(00)80588-7 9808458

